# Bioaccumulation and Phytotoxicity and Human Health Risk from Microcystin-LR under Various Treatments: A Pot Study

**DOI:** 10.3390/toxins12080523

**Published:** 2020-08-14

**Authors:** Lei Xiang, Yan-Wen Li, Zhen-Ru Wang, Bai-Lin Liu, Hai-Ming Zhao, Hui Li, Quan-Ying Cai, Ce-Hui Mo, Qing X. Li

**Affiliations:** 1 Guangdong Provincial Research Center for Environment Pollution Control and Remediation Materials, College of Life Science and Technology, Jinan University, Guangzhou 510632, China; xianglei@jnu.edu.cn (L.X.); tlyw@jnu.edu.cn (Y.-W.L.); wzrwzr@stu2018.jnu.edu.cn (Z.-R.W.); liubl2014@lzu.edu.cn (B.-L.L.); zhaohm99@jnu.edu.cn (H.-M.Z.); tlihui@jnu.edu.cn (H.L.); yingqy@jnu.edu.cn (Q.-Y.C.); 2 Department of Molecular Biosciences and Bioengineering, University of Hawaii at Manoa, Honolulu, HI 96822, USA

**Keywords:** microcystin-LR, soil pollution, input pathway, agricultural plants, bioaccumulation, degradation, phytotoxicity

## Abstract

Microcystin-LR (MC-LR) is prevalent in water and can be translocated into soil-crop ecosystem via irrigation, overflow (pollution accident), and cyanobacterial manure applications, threatening agricultural production and human health. However, the effects of various input pathways on the bioaccumulation and toxicity of MCs in terrestrial plants have been hardly reported so far. In the present study, pot experiments were performed to compare the bioaccumulation, toxicity, and health risk of MC-LR as well as its degradation in soils among various treatments with the same total amount of added MC-LR (150 μg/kg). The treatments included irrigation with polluted water (IPW), cultivation with polluted soil (CPS), and application of cyanobacterial manure (ACM). Three common leaf-vegetables in southern China were used in the pot experiments, including *Ipomoea batatas* L., *Brassica juncea* L., and *Brassica alboglabra* L. All leaf vegetables could bioaccumulate MC-LR under the three treatments, with much higher MC-LR bioaccumulation, especially root bioconcentration observed in ACM treatment than IPW and CPS treatments. An opposite trend in MC-LR degradation in soils of these treatments indicated that ACM could limit MC-LR degradation in soils and thus promote its bioaccumulation in the vegetables. MC-LR bioaccumulation could cause toxicity to the vegetables, with the highest toxic effects observed in ACM treatment. Similarly, bioaccumulation of MC-LR in the edible parts of the leaf-vegetables posed 1.1~4.8 fold higher human health risks in ACM treatment than in IPW and CPS treatments. The findings of this study highlighted a great concern on applications of cyanobacterial manure.

## 1. Introduction

Eutrophication and global warming favor frequent cyanobacterial blooms (CBs) in aquatic environments, harming environment and public health [1,2,3]. Many genera of cyanobacteria, e.g., *Microcystis*, *Oscillatoria*, and *Nostoc* can generate and then release various cyanotoxins to the environment [4,5]. Among cyanotoxins, microcystins (MCs) arouse growing global concerns owing to their ubiquity and high toxicity [3,6]. MCs are monocyclic heptapeptides and have more than 250 structural analogues, which can bind covalently to protein phosphatases 1 and 2A and induce the production of reactive oxygen species (ROS), thus leading to negative ecological effects on numerous plants and animals [7,8,9]. Epidemiologic studies showed that the increasing carcinoma incidences of liver and esophagus were correlated with chronic intake of MCs-polluted water and aquatic food [9,10,11]. Correspondingly, numerous studies regarding environmental fate and health risks of MCs in aquatic environment have been conducted over the past few decades [5,8].

Concentrations of MCs in waters are normally lower than 100 μg/L, but sometimes could be up to thousands of microgramme per litre, relying on environmental conditions and occurrence of MCs-producing cyanobacteria [8,12]. MCs in water can enter agricultural soils and show relatively high stability, because their cyclical heptapeptide structure is resistant to non-specific enzyme degradation, pH changes, and high temperature [13,14,15]. There is thus a great possibility that agricultural soils in the regions with frequent CBs outbreaks can be heavily polluted by MCs [12,16,17]. Our recent field study showed that MCs were commonly detected in agricultural soils affected by CBs in southern China, with average concentrations in the range of 3.4–15.6 μg/kg and maximum concentration up to 186.3 μg/kg [12].

MCs in soils can result in severely negative impacts on crops at perspectives of histology, cytology, and morphology, thus decreasing their growth and yield [7,18,19]. MCs are bioaccumulative in crops, posing a time- and concentration-dependent mode and raising a great concern on human health risks via the food chain [12,17,20]. Concentrations of MCs in edible parts of vegetables planted in the CBs-affected agricultural soils could be up to 382 μg/kg (fresh weight), with more than 60% of the vegetables exhibiting moderate or high human health risk via intake [12]. In addition, MCs can pose profound impacts on soil animals and microorganisms [21,22]. For example, microcystin-LR (MC-LR) caused serious oxidative stresses in tissues of earthworms and greatly affected their hatchability and survival, with medial lethal concentrations of 0.149 μg/cm and 0.460 mg/kg in filter paper test and acute soil test, respectively [21]. MC-LR suppressed soil microbial diversity and altered the dominant microbial genera of *Exiguobacterium* and *Acinetobacter* to *Prosthecobacter*, *Dechloromonas,* and *Agrobacterium* [22]. Accordingly, there are ever-increasing concerns on bioaccumulation and toxic effects of MCs in the soil ecosystem.

Main pathways of MCs entering soils from eutrophic waters include irrigation with polluted water (IPW), application of cyanobacterial manure (ACM), and overflow accident [13,23,24]. It is reasonable to expect that bioaccumulation and toxic effects of MCs vary greatly among their different input pathways from waters to soils, considering the differences of MCs in speciation and bioavailability [1,12,13]. MCs exist mainly as intracellular speciation in cyanobacterial manure and they are not bioavailable until their gradual release with lysis of cyanobacterial cells [12,25]. On the contrary, MCs in irrigation water are extracellular, and they could enter soils with repetitive irrigation and continuously exhibit bioavailability [12,16,26]. Upon overflow accident, MCs are released to soils promptly, and their bioavailability can be weakened with microbial degradation [13,22,25]. It is necessary to understand the bioaccumulation and toxicity of MCs in crops under different input pathways. However, to the best of our knowledge, the information regarding whether and how the input pathways of MCs affect their bioaccumulation in terrestrial plants and corresponding toxic effects has hardly been reported until now.

MC-LR is among the most toxic MCs and categorized as a carcinogen in group 2B by the International Agency for Research on Cancer [10,15]. In the present study, MC-LR was selected as the target compound to conduct pot experiments using different treatments at the same total amount fortified. These treatments included IPW, cultivation with polluted soil (CPS), and ACM, which were used to simulate various input pathways of MC-LR entering agricultural soils, i.e., irrigation, overflow accident, and cyanobacterial manure. Three typical leaf vegetables widely planted in southern China, namely *Ipomoea batatas* L. (sweet potato), *Brassica juncea* L. (leaf mustard), and *Brassica alboglabra* L. were used in the present study. The aims of the present study were to compare the effects of three input pathways on bioaccumulation and corresponding toxic effects of MC-LR in leaf vegetables, MC-LR degradation in soils, and human health risks of MC-LR via consumption of the tested vegetables. The findings of the study provided insights into how the input pathways affect bioavailability of MCs in soil-plant ecosystem.

## 2. Results and Discussion

### 2.1. Microcystin-LR (MC-LR) Bioaccumulation in Vegetables under Various Treatments

**Figure 1:**
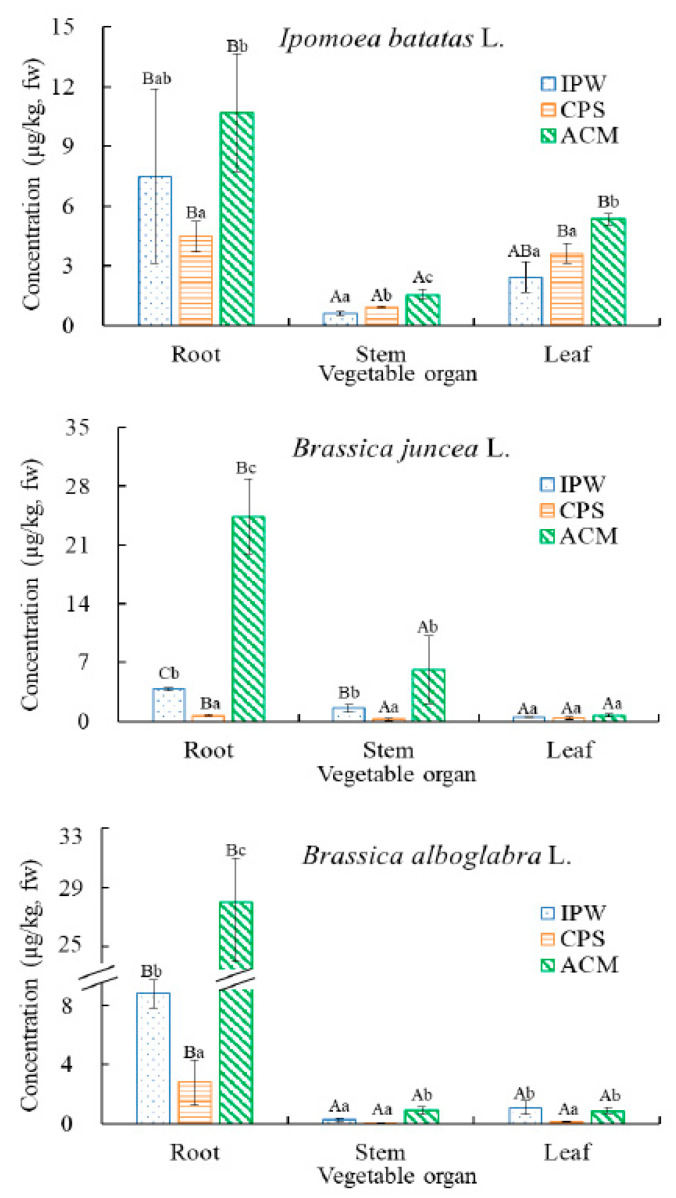
Bioaccumulation of microcystin-LR (MC-LR) in different parts of three tested vegetables upon various treatments. IPW, CPS, and ACM stand for irrigation with polluted water, cultivation of polluted soil, and application of cyanobacterial manure, respectively. The same capital letter in each treatment indicates no significant difference in MC-LR concentration among different vegetable parts (*p* > 0.05). The same low case letter in each vegetable tissue indicates no significant difference in MC-LR concentration among different treatments (*p* > 0.05). These annotations mentioned above are no longer annotated hereinafter.

Although quite a few papers have reported bioaccumulation of MCs in terrestrial plants [4,17,27,28], they are hardly related to the effects of various input pathways on the bioaccumulation of MCs. In the present study, bioaccumulation of MCs was observed in roots, stems, and leaves of the tested vegetables under various input pathways, with MC-LR concentrations in *I. batatas*, *B. juncea*, and *B. alboglabra* ranging from 0.63 to 10.7 μg/kg, 0.24 to 24.4 μg/kg and 0.1 to 15.0 μg/kg, respectively (Figure 1). Among the input pathways, the concentrations of MC-LR in most organs of the three tested vegetables in ACM treatment were significantly higher than those in IPW and CPS treatments (*p* < 0.05) up to 37.2 times, although there were comparable cases observed between ACM and IPW treatments in root of *I. batatas* and leaf of *B. juncea* and *B. alboglabra* (Figure 1). ACM treatment showed generally higher bioaccumulation of MC-LR in the vegetables than IPW and CPS. As for IPW and CPS treatments, the former generally showed higher MC-LR bioaccumulation than the latter, with significant cases observed in root and stem of *B. juncea* and root and leaf of *B. alboglabra* (*p* < 0.05, Figure 1).

Varying bioavailability and corresponding degradation capacity of MC-LR could account for the differences in MC-LR bioaccumulation among different treatments. In ACM treatment, MC-LR is mainly stored in cyanobacterial cells as intracellular-MCs, and the cyanobacterial cell may protect MCs from rapid degradation in soils [12,25]. With lysis of cyanobacterial cell, MC-LR could be gradually released and continually taken up by the vegetables, thus showing high bioaccumulation. In IPW treatment, MC-LR exhibited as dissolved state and had relatively higher degradation capacity in soils, but the repetitive irrigation offset MC-LR loss and made it show higher bioaccumulation in the vegetables [12,16]. As for CPS treatment, MC-LR was subject to continuous degradation and loss once it entered soils [21,25], thus likely displaying a lower residual content in soils and corresponding lower bioaccumulation in the vegetables. On the other hand, MC-LR can be also metabolized in plants via a glutathione (GSH) pathway, in which MC-LR can react with GSH and form GSH-MC-LR conjugates via catalysis of glutathione transferase [4,26]. Depuration rates of MC-LR in lettuce and spinach were determined at 9.5 and 8.1 μg/kg/d (dry weight), respectively [26]. In CPS treatment, MC-LR was taken up and bioaccumulated in the vegetables, but it simultaneously underwent a depuration process. The depuration rate of MC-LR can be close to and even exceed its uptake rate in plants with increasing MC-LR degradation in soils, thus leading to a lower MC-LR bioaccumulation in CPS treatment. On the contrary, the gradual release of MC-LR in ACM treatment or the repetitive input of MC-LR in IPW treatment could maintain a higher MC-LR residual content in the soils and a corresponding greater uptake rate in the vegetables. Therefore, although depuration process of MC-LR also occurred in vegetables of ACM and IPW treatments, uptake rate of MC-LR can exceed its depuration rate, leading to higher MC-LR bioaccumulation in the two treatments. Further studies should be conducted to understand time-dependent uptake and depuration of MC-LR and corresponding mechanisms in various terrestrial plants under different input pathways.

### 2.2. Uptake and Transfer of MC-LR in Vegetables under Various Treatments

Concentrations of MCs in roots were 1–2 orders of magnitude higher than those in stems and leaves (Figure 1). Meanwhile, MC-LR was not detected in the vegetables of control treatments without addition of MC-LR, denying the possibility of MC-LR uptake via leaf stomata of vegetables. The stomata uptake of MC-LR by the plants was also not observed even at the condition of using drip or spray irrigation [17,24]. Accordingly, MC-LR was absorbed mainly via the vegetable roots, with its subsequent translocation driven by transpiration stream [16,17].

Concentrations of MC-LR in the vegetables depended on species and organs of the vegetables and the input pathways of MC-LR. To be specific, higher MC-LR concentration in root was observed in *B. juncea* (24.4 μg/kg) than *I. batatas* (10.7 μg/kg) and *B. alboglabra* (15.0 μg/kg) in ACM treatment, but this trend was opposite in IPW and CPS treatments (Figure 1). Concentrations of MC-LR in the stems of *B. juncea* in ACM and IPW treatments were one order of magnitude higher than those of *I. batatas* and *B. alboglabra.* The MC-LR concentrations in *B. juncea* in CPS treatment were comparable with those of *B. alboglabra* and 3-fold lower than those of *I. batatas* (Figure 1). Concentrations of MC-LR in the leaves of *I. batatas* were one order of magnitude higher than those of the other two vegetables no matter which treatments were (Figure 1). This indicated higher translocation and bioaccumulation capacities of MC-LR in edible parts of *I. batatas*, relative to *B. juncea* and *B. alboglabra*.

To quantitatively appraise the uptake and translocation of MC-LR in the tested vegetables using various input pathways, the root concentration factor (*RCF*), root translation factor (*RTF*), and stem translation factor (*STF*) were calculated, according to Equations (1)–(3) listed in Materials and Methods. *RCF* values of MC-LR in the tested vegetables significantly decreased in the order of ACM > IPW > CPS (*p* < 0.05, Table 1). This indicated that ACM enhanced MC-LR bioconcentration in the vegetable roots, which was likely associated with gradual release of MC-LR and its continuous uptake by the vegetables [12,25]. On the contrary, *RTF* and *STF* values of MC-LR in the vegetables varied little among various input pathways of MC-LR (*p* > 0.05, Table 1). Accordingly, MC-LR bioaccumulation in the vegetables in ACM was enhanced by root bioconcentration capacity rather than root/stem translocation capacity.

Compared with *RCF* value, *RTF* and *STF* values of each vegetable were generally one and two orders of magnitude higher, respectively (Table 1). The recent field study also showed high bioconcentration factors (3.8–23.4) of MCs in edible parts of various leaf vegetables including celery, lettuce, cabbage, and garlic chives [12]. These indicated MC-LR could be readily translocated into the edible parts of leaf vegetables once it was absorbed by the vegetable roots. Consequently, root uptake acted as a key factor determining MC-LR bioaccumulation in the leafy vegetables, which explained higher MC-LR bioaccumulation in ACM treatment via enhancing the root bioconcentration. Considering the degradation of MC-LR in soils [21,25], its *RCF* values could be underestimated when they were calculated with the total fortified amount of MC-LR in soils based on Equation (1). Such a calculation is still acceptable for comparing the root uptake capacity of MC-LR among different input pathways. This is because complex and varying biochemical processes of MC-LR happen after it enters soils by different pathways. The processes include gradual release and corresponding slow degradation of MC-LR in ACM treatment, repetitive input and corresponding degradation of MC-LR in IPW treatment, and relatively rapid MC-LR degradation in CPS treatment. It is difficult to identify how much of the added amount of MC-LR is actually involved with the root uptake, and thus the total added amount of MC-LR is used for calculation of RCF values.

In terms of various vegetables, STF values of *Ipomoea batatas* L. were significantly higher than those of *Brassica juncea* L. in each treatment, with difference up to 16.9 times (Table 1). Meanwhile, MC-LR concentrations in the edible parts (leaves) of *Ipomoea batatas* L. were significantly higher than the other two vegetables (*p* < 0.05, Figure 1). Therefore, *Ipomoea batatas* L. showed higher bioaccumulation capacity of MC-LR in the edible parts relative to the other two vegetables, although its STF values were comparable with those of *Brassica alboglabra* L. (*p* > 0.05). It is thus not recommendable to grow *Ipomoea batatas* L. in the soils affected by CBs, because of its higher bioaccumulation capacity of MC-LR and corresponding higher potential human health risk via diet.

### 2.3. MC-LR Degradation in Soils under Various Treatments

**Figure 2:**
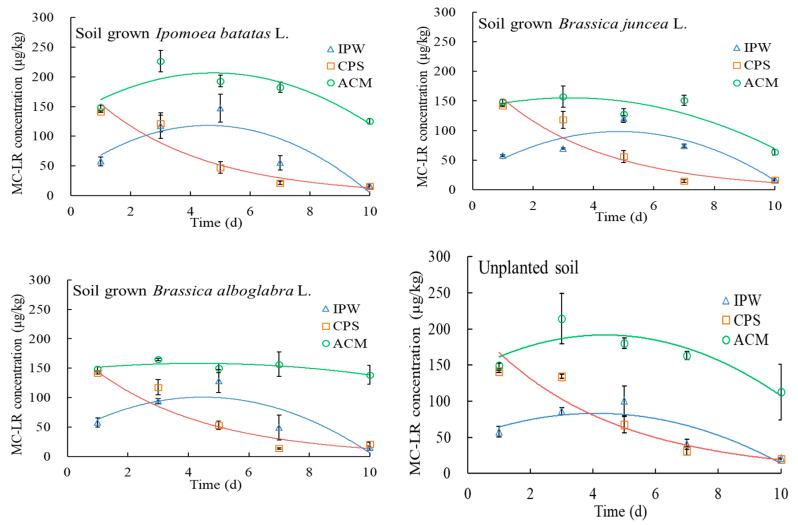
MC-LR degradation in soils of various treatments.

There were different concentration profiles of MC-LR in soils with increasing cultivation time in various input pathways (Figure 2). Soil MC-LR concentrations in both ACM and IPW treatments increased first, and then decreased with the increasing cultivation time. Soil MC-LR concentrations in CPS treatment decreased continuously (Figure 2). The gradual release of MC-LR in ACM treatment and repetitive input of MC-LR in IPW treatment [12,25,26], together with the subsequent microbial degradation of MC-LR in soils, could account for the characteristic concentration profiles of soil MC-LR in the two treatments. Because two different processes could change soil MC-LR concentrations in the ACM treatment (release + degradation) and IPW treatment (input +degradation), a mono basic quadratic equation was used to fit their data of soil MC-LR concentrations. As for CPS treatment, an exponential equation was used to fit the data of soil MC-LR concentrations. Satisfactory determining factors (*R*^2^ > 0.7) with *p* < 0.05 indicated a good match with the fitted equations (Table 2).

A half-life (*t*_1/2_) of MC-LR was defined as a cultivation time when MC-LR concentration in soil was decreased to half of the total MC-LR added amount, i.e., 75 μg/kg. According to the fitted equations, half-lives of MC-LR in soils of various treatments were obtained (Table 2). The half-lives of MC-LR in the ACM treatments were 1.3–2.2 and 2.6–4.6 fold longer than those in IPW and CPS treatments, respectively. After 10-days cultivation, more than 75% of total MC-LR added amount remained in the soils, except those planted *B. juncea* (42.9%) (Figure 2). These indicated that ACM limited MC-LR degradation in soils. The lower degradation and higher residual concentrations of MC-LR in the ACM soils could favor its root bioconcentration and corresponding bioaccumulation in the vegetables, as observed in Figure 1 and Table 1. As for IPW treatment, the repetitive input rather than one-time input could avoid rapid MC-LR degradation in the soils, thus half-lives of MC-LR in the treatment were 1.4–2.2 fold longer than those of CPS treatment (Table 2). However, MC-LR residuals in soils of IPW treatment decreased rapidly with increasing cultivation time (Figure 2). After 10-days cultivation, only ~10% of total MC-LR added amount was kept in the soils of IPW treatment (Figure 2), similar to CPS treatment. This may indicate that repetitive input of MC-LR via irrigation water was conducive to MC-LR-degrading bacteria, and thus enhanced MC-LR degradation with increasing cultivation time [22].

Compared to CPS treatment without plantation, the CPS treatments with plantation showed ~1.2-fold shorter half-life of MC-LR and 1.0–1.3 fold lower MC-LR residual after 10-days cultivation (Table 2), indicating enhanced MC-LR degradation in soils by plantation. Uptake amount of MC-LR (vegetable MC-LR concentration × vegetable weight) in all vegetables accounted for only 0.001–0.05% of total amount of added MC-LR in soils (Figure 2 and Table 3). This could indicate that enhanced microbial degradation rather than plant uptake played a key role in increased MC-LR degradation in soils with plantation, relative to the soils without plantation in CPS treatment. As for IPW treatment after 10-days cultivation, 1.2–1.3 fold longer half-life and 1.2–1.5 fold lower residual of MC-LR were observed in soils with plantation than without plantation (Table 2 and Figure 2). This can be associated with the adaptation period between the planted vegetables and the MC-LR-degrading bacteria stimulated by IPW [29,30]. Such an adaptation period prolonged the half-lives of MC-LR in soils of IPW treatments, but the degradation rates of MC-LR increased rapidly after the adaptation period (i.e., after 6-days cultivation, Figure 2), thus leading to a low MC-LR residual in soils after 10-days cultivation. In the terms of ACM treatments, the effects of plantation on MC-LR degradation were vegetable-dependent. Compared to the ACM treatment without plantation, only plantation with *B. juncea* was conducive to MC-LR degradation, while *I. batatas* and *B. alboglabra* showed no favored and even unfavourable effects on MC-LR degradation in soils (Table 2 and Figure 2).

Two groups of bacteria degraded MC-LR in soils of ACM treatments. The lytic-cyanobacteria bacteria, including *Arthrobacter* sp., *Oxalobacteraceae* sp. and *Pedobacter* sp., can restrain cyanobacteria growth or lyse the cells via releasing extracellular enzymes and algal-lytic compounds [31,32]. The second group is MC-degrading bacteria that include the genera *Sphingopyxis* and *Sphingomonas*. This group of bacteria contain a *mlrBDAC* gene capable of degrading MC analogues [15,33]. A recent study reported a complete biodegradation pathway of MC-LR by an indigenous bacterium, i.e., *Sphingopyxis* sp. YF1, in which MC-LR was gradually degraded to linear MC-LR, tetrapeptide, 3-amino-9-methoxy-2, 6, 8-trimethyl-10-phenyl-deca-4, 6-dienoic acid (Adda) and phenylacetic acid acting as precursor of acetyl-CoA [15]. It was noteworthy that a single *Acinetobacter* sp. or a single enzyme (Microcystinase A) was identified to degrade MC-LR and inhibit growth of *Microcystis aeruginosa* simultaneously [14,34]. The specific distribution of lytic-cyanobacteria bacteria and MC-LR-degrading bacteria accounts for the vegetable-dependent MC-LR degradation in soils of ACM treatments. Further studies are recommended to identify which genera of bacteria and corresponding key genes are responsible for MC-LR degradation in soil-plant ecosystem under different input pathways.

### 2.4. Toxicity to Vegetables from MC-LR under Various Treatments

**Figure 3:**
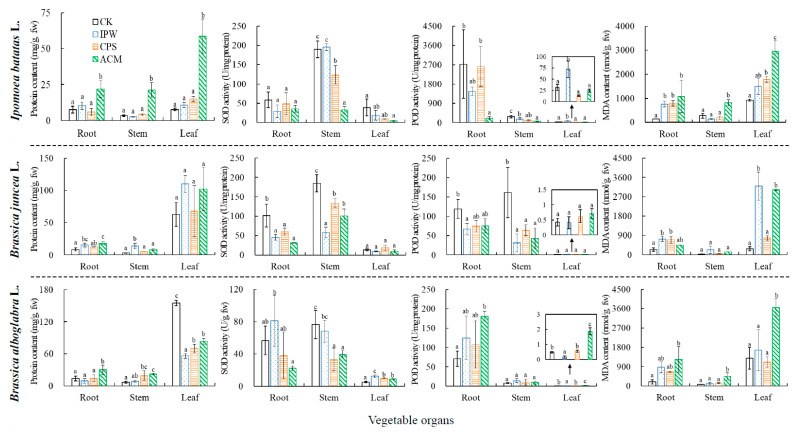
Contents of protein and malondialdehyde (MDA) and activities of superoxide dismutase (SOD) and peroxidase (POD) in three tested vegetables in various treatments. The same letters in each vegetable tissue indicates no significant difference (*p* > 0.05). CK stands for the control.

MCs, especially MC-LR, can induce reactive oxygen species (ROS) and cause oxidative damage to terrestrial plants [7,18,35]. In the present study, the effects of MC-LR on content of total protein (TP) and malondialdehyde (MDA), activities of antioxidant enzymes (superoxide dismutase, SOD; peroxidase, POD), and biomass indicators (plant height, main root length, total weight, and aerial part weight) were investigated. Such effects of MC-LR varied with the toxicity indicators, input pathways of MC-LR and species, and organs of the vegetables (Figure 3 and Table 3). Generally, MC-LR in ACM treatment significantly increased TP contents in all the organs of the vegetables (*p* < 0.05), but MC-LR in CPS and IPW treatments caused little change in the TP contents (*p* > 0.05). An exception was observed in *B. alboglabra* leaf where MC-LR in all treatments significantly inhibited TP contents (*p* < 0.05). The increased TP contents by MC-LR in ACM treatment could be associated with activation of some functional enzymes, e.g., glutathione *S*-transferase (GST) and glutathione reductase (GR) [4,26,36]. Plant GST could be activated by MC-LR exposure, which catalyze formation of glutathione-MC-LR complex as the first step of MC-LR detoxification [26,37,38]. To maintain glutathione pool expended by MC-LR, plant GR are usually activated by MC-LR exposure [4,26]. On the other hand, the decreased TP content in the leaf of *B. alboglabra* can be associated with damage of the antioxidative enzymes by the MC-LR exposure [4,26,36].

Plant SOD and POD can effectively remove superoxide anions (·O_2_^−^) and hydrogen peroxide (H_2_O_2_) induced by MC-LR, thus protecting plant cell from oxidative damage [18,36]. In the present study, MC-LR posed generally significant inhibition on activities of SOD and POD in roots and stems of the vegetables (*p* < 0.05), with higher inhibition rates in ACM treatment (32.7~92.3%) than in IPW (10.8~68.7%) and CPW treatments (16.7~60.1%, Figure 3). The decreased activities of SOD and POD were likely because the excessive ROS induced by MC-LR damaged their structures [18]. It was different from roots and stems of the vegetables that their leaves were observed with complex situations regarding the effects of MC-LR on activities of SOD and POD. For example, MC-LR in ACM treatment caused little influence on activities of SOD and POD in leaf of *B. juncea*, but significant inhibition on SOD activity in leaf of *I. batatas* (*p* < 0.05) and significant activation on activities of POD and SOD in leaf of *B. alboglabra* (*p* < 0.05). Varying responses of SOD and POD to MC-LR exposure reflected their different scavenging activities of ·O_2_^-^ and H_2_O_2_ in various vegetable leaves. Besides antioxidant enzyme, non-enzymatic antioxidant substance in plant can play an important role in detoxification of MC-LR. For example, phenolic compounds widely exist in plants [39]. They have powerful antioxidant capacity and can protect plant cell from oxidative damage [39]. The different toxicity responses of various vegetables to MC-LR stress could be related to different content of these non-enzymatic antioxidant substances, e.g., phenolic compounds (Figure 3).

MDA in organisms was formed by the peroxidation reaction between ROS and unsaturated fatty acids in cellular membranes, which was considered as an important index for intracellular oxidative damage [21,40]. In the present study, MC-LR in all treatments generally resulted in significant increase of MDA contents, with higher increase rates in ACM treatment (1.8–10.9 fold) than in IPW (0.5–11.9 fold) and CPW treatments (0.7–5.3 fold, Figure 3). The increase of MDA content indicated that antioxidant systems of the vegetables failed to eliminate the ROS induced by MC-LR, thus causing oxidative damage. The highest contents of MDA and TP but lowest activities of SOD and POD observed in the vegetables of ACM treatment indicated that ACM caused the greatest oxidative stresses in the vegetables among the different MC-LR treatments.

As expected, MC-LR in ACM treatment caused the greatest inhibition on biomasses of the vegetables, with inhibition rates of 22.5~43.7%, 34.0~60.0%, 45.0~73.6%, and 47.5~74.2 for plant height, main root length, total weight, and aerial part weight, respectively (Table 3). The inhibition rates of MC-LR in ACM treatment on all the biomasses indicators were about 1.2–2 fold higher than those in IPW and CPS treatments (Table 3). The high inhibition of vegetable biomasses observed in ACM treatment indicated great phytotoxicity of MC-LR in the treatment. Based on Pearson correlation analyses, there were negative correlations between biomasses indicator values and both MC-LR concentrations and MDA contents of the vegetables, but positive correlations between MC-LR concentrations and MDA contents (Tables S1–S3). Taking *I. batatas* as an example, root MC-LR concentration, stem MC-LR concentrations, stem MDA contents, and leaf MDA contents showed significantly negative correlations with main root length and plant height (*p* < 0.05); but there were significantly positive correlations between root MC-LR concentrations and leaf MDA contents, between stem MC-LR concentration and stem and leaf MDA content, as well as between leaf MC-LR concentrations and leaf MDA content (*p* < 0.05, Table S1). These indicated that MC-LR bioaccumulation can cause great phytotoxicity by inducing oxidative damage. Accordingly, ACM could cause high toxicity to the vegetables, mainly due to high MC-LR bioaccumulation observed in the treatment (Figure 1), although other bioactive compounds could be contained in the ACM treatment, microcystin-RR and microcystin-YR [13,28].

### 2.5. Health Risk from Consuming the Vegetables under Various Treatments

MCs bioaccumulation poses a threat to human health via diet [12,16,17]. To assess the health risk of MC-LR via consuming the edible parts (leaves) of the planted vegetables, the estimated daily intake (*EDI*) and risk quotient (*RQ* = *EDI*/*RfD*) were calculated. *EDI* values and *RQ* values of MC-LR in ACM treatment ranged, respectively, from 0.003 to 0.022 μg/kg/d and 0.09 to 0.55, both of which were 1.1~4.8 folds higher than those in IPW and CPS treatments (Table 4). A widely-acceptable criterion for evaluating health risk was used in this study, namely, high health risk (*RQ* > 1), moderate health risk (0.1 ≤ *RQ* ≤ 1), and low health risk (*RQ* < 0.1) [12,41]. In ACM treatments, the consumption of *I. batatas* and *B. alboglabra* showed medium health risk; while in IPW or CPS treatments, just consumption of *I. batatas* displayed medium health risk; in the other situations, only low health risks were estimated (Table 4). This indicated that ACM could likely result in higher health risks of consuming the vegetables relative to IPW and CPS, although significant difference in RQ value was only observed between CPS and ACM of *Brassica alboglabra* L. (*p* < 0.05). The situation can be much worsened when common co-existence of multiple MC analogues in the cyanobacterial manure is considered [12,13] and they could also pose severe health risks during ACM. Injection of MCs can cause severe multiple organ injuries and even stimulate the expression of proto-oncogenes and oncogenes cytokines in liver and colon, thus enhancing the cancer incidences [10,11,42]. Therefore, ACM is not recommended for soil fertility improvement, especially those affected by CBs, which may cause higher MCs bioaccumulation and corresponding greater phytotoxicity and higher human health risks.

## 3. Conclusions

Various input pathways of MC-LR can result in its bioaccumulation in edible parts of leaf vegetables from soils, including irrigation with polluted water (IPW), cultivation of polluted soil (CPS), and application of cyanobacterial manure (ACM). ACM treatment causes higher bioaccumulation of MC-LR relative to IPW and CPS treatments. This is associated with limited degradation and high residual content of MC-LR in soils of ACM treatment. Such a high bioaccumulation of MC-LR in ACM treatment may cause high phytotoxicity due to oxidative damage and great human health risks via diet. The findings of this study raise great concerns about MCs entering terrestrial ecological system by different input pathways, especially by ACM. Further studies should be conducted to reveal microbial populations and key genes responsible for MCs degradation in soils and their metabolism in terrestrial plants under different input ways.

## 4. Materials and Methods

### 4.1. Materials

MC-LR standard was bought from Taiwan Algal Science, Inc. Fresh cyanobacterial bloom was collected from Lake Dianchi that is a typical eutrophic water in Yunnan province of southern China, usually containing MCs-producing *Microcystis viridis* (61.9%) and non-toxic *Microcystis wesenbergii* (24.8%) [12]. Cyanobacterial manure was obtained from the collected cyanobacterial bloom by freeze-drying and then grinding. Concentration of MC-LR in the cyanobacterial manure was detected as 50 ± 8.7 μg/g (dry weight) using a HPLC-MS/MS method based on our recent literature [12]. Agricultural soil (0~20 cm) used in this study was obtained from an organic farm in Guangzhou, China, with no MC-LR detected. Its basic physicochemical properties were measured according to a recommended method [43], including pH (7.5), organic matter (43.9 g/kg), cation exchange capacity (16.0 cmol/kg), and soil texture (36.9% of sand, 40.5% of silt, and 22.7% of clay). Seeds of *Ipomoea batatas* L., *Brassica juncea* L., and *Brassica alboglabra* L. were obtained from Guangdong Academy of Agricultural Science. Ethylene diamine tetraacetic acid (EDTA) and sodium pyrophosphate were purchased from Guangzhou Chemical Reagent Co., Ltd. (Guangzhou, China). HPLC-grade methanol and trifluoroacetic acid were bought from Sigma-Aldrich (Steinheim, Germany). Ultrapure water was prepared by a Unique-R20 equipment purchased from Research Scientific Instruments Corporation, Xiamen, China.

### 4.2. Methods

#### 4.2.1. Pot Design

Based on actual concentration of MCs in agricultural soils [12], three types of treatments (including IPW, CPS, and ACM) with a same added amount (150 μg/kg) of MC-LR were set to investigate bioaccumulation, toxicity, and health risk of MC-LR in the selected leaf vegetables. In IPW and CPS treatments, MC-LR solution prepared by adding MC-LR standard to ultrapure water was used. In brief, 100 mL of MC-LR solution (0.525 mg/L) was irrigated into per pot per day for IPW treatment. Such an irrigation was repeated for 10 days continuously. Five-hundred milliliters of MC-LR solution (1.05 mg/L) was spiked into per pot at one time for CPS treatment. As for ACM treatment, 10.5 g of the prepared cyanobacterial manure (50 μg/g of MC-LR) was added into per pot at one time and then homogenized. The treatment without addition of MC-LR was set as control. Each treatment was conducted in quadruplicate.

Polypropylene pots with ~20 cm of inner diameter and ~18 cm of height were used in this study. Each pot was loaded with 3.5 kg of soils containing 8 g of base fertilize, i.e., monopotassium phosphate and urea at mass ratio of N/P/K of 4:3:4. Before pot cultivation, plant seeds were germinated and incubated in a nutrient substrate prepared from unpolluted plant straw compost and soils [44]. To evaluate the uptake and translocation of MC-LR in mature plant, plants incubated for 40 days were used in this study, with three plants per pot. The pot experiments were carried out for 10 days in a greenhouse at natural temperature (24~32 °C) in April 2016, with all the pots randomly arranged. Deionized water was used to keep moisture content of the soils, based on the evaporation capacity. During the pot experiments, soil samples were collected for all treatments at days 1, 3, 5, 7, and 10, respectively. The collected soil samples were freeze-dried, ground, and then filtered (0.42 mm) for analysis of MC-LR. After 10 d of cultivation, all the plants were collected and then washed by deionized water to remove the adhesive soil particles. The biomass indicators of the collected plant samples were measured after drying by tissue paper, including height, root length, and fresh weight. Afterwards, all the plant samples were cut into three parts, i.e., root, stem, and leaf. Each part of the plant samples was divided into two portions for analysis of MC-LR and determination of antioxidant activities and MDA, respectively. The portion used for MC-LR analysis was pretreated as mentioned above for the soil samples.

#### 4.2.2. Extraction and Analysis of MC-LR in Soil and Vegetable Samples

Based on our developed methods [45,46], extraction of MC-LR in both soil and vegetables was conducted by ultrasonic extraction, followed by solid-phase extraction cleanup using C_18_ cartridge. The extractants used for extraction of the soil and vegetables were 0.1 M EDTA-sodium pyrophosphate solution and acidified methanol solution (methanol/water/trifluoroacetic acid at 80/19.9/0.1, *v*/*v*/*v*), respectively. Analysis of MC-LR was performed on an alliance 1100 liquid chromatograph (Agilent, Palo Alto, CA, USA) interfaced with an API 4000Q-Trap triple-quadrupole mass spectrometer (Applied Biosystems, Foster city, CA, USA), with 5 µL of injection volume. The mass spectrometer was set in positive mode electrospray ionization with multiple-reaction monitoring mode. Separation of MC-LR was used by Eclipse Plus C18 column (150 mm × 2.1 mm i.d., 5 µm). Mobile phases A and B were selected as acetonitrile and 0.2% formic acid solution (*v*/*v*), with flow rate at 0.3 mL min^−1^. In the gradient program, mobile phase A was linearly enhanced from 20% to 80% in 2 min (kept for 4.5 min) and then returned to 20% within 0.1 min (kept for 9.4 min). Retention time of MC-LR in the gradient program was 5.33 min, and it was quantified by selected reaction monitoring, with precursor ion of 996.1 [M+H]^+^, production ion of 135/213, decluster potential of 96/91, and 100/85 of collision energy voltage. For ease of evaluating risk calculation, the detected concentration of MC-LR in the freeze-dried vegetable sample was converted to the concentration in the fresh weight, based on water content of the fresh vegetables (Table S4).

In the analytical conditions used in this study, the limit of quantification of MC-LR, i.e., a concentration producing 10-fold signal-to-noise was detected as 0.25 ng/g (fw) and 1.6 ng/g (dw) for soil and vegetable sample, respectively. The spiked recoveries of MC-LR at 5 ng/g were in the range of 72.6~97.4% and 61.3~107.9% for soil and vegetable samples, respectively (Table S5). During analysis of MC-LR in the samples, a spiked sample at 5 ng/g, a procedural blank, and a random duplicate sample were determined with each batch of seven samples to ensure the analytical quality.

#### 4.2.3. Determination of Antioxidant Enzymes and MDA Content

Fresh plant sample (0.2 g) was fully ground at ice-bath in the presence of 10 mL of phosphate buffer solution (pH = 7.4). The sample homogenate was centrifuged for 10 min at 5000 rmp/min and 4 °C to obtain sample supernatant. The contents of TP and MDA and activity of SOD were determined using a UV-2450 spectrophotometer (Shimadzu, Japan), based on instructions of testing kits purchased from Nanjing Jiancheng Bioengineering Institute, China [47]. Activity of POD was determined using a guaiacol method, in which POD catalyzed oxidation of guaiacol by H_2_O_2_ to form a dark brown compound having a visible response at 470 nm [48]. A 0.01-unit change in absorbance of formed dark brown compound at 470 nm within 20 s was recorded as one unit of POD activity [48].

#### 4.2.4. Data Processing

Root concentration factor (*RCF*) and translation factor (*TF*) of MC-LR in the tested vegetable were calculated without considering its degradation and metabolism, according to the following equations:

*RCF* = *c_r_*/*c_s_* (1)

*RTF* = *c_st_*/*c_r_* (2)

*STF* = *c_s_*/*c_l_* (3)

where *c_s_* indicates total MC-LR amount added into soil (150 μg/mg); *c_r_*, *c_st_*, and *c_l_* indicate MC-LR concentration in root, stem, and leaf of vegetable sample, respectively; *RTF* and *STF* indicate translation factor of ML-LR from root to stem and from stem to leaf, respectively.

Health risk quotient (*RQ*) via consumption of the vegetable was evaluated according to the following Equations (4) and (5):

*RQ* = *EDI*/*RfD* (4)

*EDI* = (*c_l_* × *DC*)/*BW* (5)

where *EDI* indicates estimated daily intake, RfD is daily reference dose (0.04 μg/kg/d) of MC-LR set by World Health Organization, *c_l_* has been defined in Equation (3), *DC* and *BW* indicate daily consumption of vegetable (335.5 g) and average body weight (65 kg) of Chinese adult, respectively, whose values were obtained from Exposure Factors Handbook of Chinese Population [49]. Besides tuberous root or seed, leaves of *I. batata* and *B. juncea* are popular food in Asian countries, especially China [50,51]. Because no tuberous root or seed of *I. batata* and *B. juncea* were generated in the pot experiments, leaves of the tested vegetables were selected as the focused edible parts for conducting health risk assessment.

In addition, basic statistics including calculation of mean and standard deviation (SD) were finished by Microsoft Excel 2013 (Microsoft Co., Redmond, WA, USA). Pearson correlation, principal component analysis (PCA), and one-way analysis of variance (ANOVA) followed by a Duncan’s (D) test were performed on SPSS 21.0 (SPSS Inc., Chicago, IL, USA). During ANOVA analysis, the Dunnett’s T3 (3) test was used when equal variance assumption was invalid based on Levene’s test of equality of error variances. Statistical significance was defined at *p* < 0.05.

## Figures and Tables

**Table 1:** Root concentration factor (*RCF*), root translation factor (*RTF*), and stem translation factor (*STF*) of MC-LR in three tested vegetables in various treatments.

Vegetable	Treatment	*RCF*	*RTF*	*STF*
*Ipomoea batatas* L.	IPW	0.05 ± 0.02 Aab	0.12 ± 0.08 Ba	4.07 ± 1.54 Ba ^a^
	CPS	0.03 ± 0.01 Ba	0.21 ± 0.02 Ca	3.94 ± 0.39 Ba
	ACM	0.07 ± 0.02 Ab	0.15 ± 0.03 Ba	2.87 ± 1.20 Ba
*Brassica juncea* L.	IPW	0.03 ± 0.01 Ab	0.41 ± 0.11 Ca	0.31 ± 0.11Aa
	CPS	0.004 ± 0.001 Aa	0.36 ± 0.07 Ba	1.90 ± 1.12 Ab
	ACM	0.16 ± 0.02 Bc	0.25 ± 0.12 Ba	0.17 ± 0.01Aa
*Brassica alboglabra* L.	IPW	0.06 ± 0.01 Ab	0.03 ± 0.01 Aa	2.62 ± 1.80 Ba
	CPS	0.02 ± 0.01 Ba	0.02 ± 0.01 Aa	2.73 ± 0.80 ABa
	ACM	0.19 ± 0.02 Bc	0.03 ± 0.01 Aa	1.03 ± 0.45 ABa

^a^ The same small letters in each factor of one vegetable under different treatments or the same capital letters in each factor of one treatment using different vegetables indicate no significant differences (*p* > 0.05).

**Table 2:** The fitted equations for MC-LR degradation in soils grown in different vegetables.

Vegetable	Treatment	Equation ^a^	*R* ^2^ ^b^	Half-life (*t*_1/2_, d) ^c^
*Ipomoea batatas* L.	IPW	y = −3.9x^2^ + 35.6x + 36.5	0.744 *	7.7
	CPS	y = 203.1e^−0.28x^	0.936 **	3.6
	ACM	y = −3.15x^2^ + 30.0x + 135.1	0.786 **	11.3
*Brassica juncea* L.	IPW	y = −3.1x^2^ + 30.1x + 25.4	0.832 **	7.6
	CPS	y = 203.9e^−0.28x^	0.863 **	3.6
	ACM	y = −1.9x^2^ + 12.0x + 136.0	0.827 **	9.8
*Brassica alboglabra* L.	IPW	y = −3.1x^2^ + 27.9x + 38.4	0.750 *	7.4
	CPS	y = 187.3e^−0.26x^	0.792 **	3.5
	ACM	y = −0.61x^2^ + 5.2x + 146.9	0.643 *	16.0
Non-planted vegetable	IPW	y = −2.0x^2^ + 16.2x + 50.0	0.726 *	6.1
	CPS	y = 213.4e^−0.24x^	0.947 **	4.3
	ACM	y = −2.7x^2^ + 23.2x + 140.8	0.800 **	11.4

^a^ “x” and “y” in the equation indicate cultivation days and MC-LR concentration in soil, respectively. ^b^ “*” and “**” indicate *p* < 0.05 and *p* < 0.01, respectively. ^c^ Half-life indicates a cultivation time when MC-LR concentration in soil was decreased to half of the total amount of added MC-LR, i.e., 75 μg/kg.

**Table 3:** Biomasses of the tested three vegetables in various treatments.

Vegetable	Treatment	Plant Height (cm)	Main Root Length (cm)	Total Weight (g/plant)	Aerial Part Weight (g/plant)
*Ipomoea batatas* L.	CK ^a^	69.5 ± 7.8 a ^c^	25.5 ± 0.7 b	26.4 ± 2.6 b	25.1 ± 2.3 b
	IPW ^b^	67.7 ± 18.0 a	24.0 ± 1.0 b	16.4 ± 0.1 a	15.5 ± 0.9 a
	CPS	69.0 ± 5.7 a	25.0 ± 0.1 b	15.5 ± 3.2 a	15.1 ± 4.2 a
	ACM	53.8 ± 5.6 a	16.8 ± 1.9 a	14.3 ± 3.4 a	13.1 ± 3.7 a
*Brassica juncea* L.	CK	58.0 ± 10.2 b	21.7 ± 8.1 b	55.6 ± 5.2 b	54.1 ± 3.3 c
	IPW	39.7 ± 6.1 a	10.0 ± 1.0 a	39.8 ± 8.7 a	37.6 ± 7.9 ab
	CPS	46.7 ± 7.5 ab	11.0 ± 2.7 a	46.0 ± 2.0 ab	39.6 ± 0.8 b
	ACM	32.7 ± 4.7 a	8.7 ± 0.6 a	30.6 ± 7.5 a	28.4 ± 0.4 a
*Brassica alboglabra* L.	CK	44.0 ± 7.0 c	13.0 ± 6.1 b	35.4 ± 4.6 c	34.5 ± 4.5 c
	IPW	34.3 ± 1.8 b	8.0 ± 0.1 a	17.6 ± 5.5 b	15.6 ± 7.5 ab
	CPS	35.0 ± 1.4 ab	10.5 ± 2.1 ab	16.5 ± 2.2 b	15.9 ± 2.2 b
	ACM	25.6 ± 1.4 a	8.7 ± 0.6 ab	9.4 ± 0.9 a	8.9 ± 0.9 a

^a^ CK indicates control treatment without addition of MC-LR. ^b^ IPW, CPS, and ACM indicate irrigation of polluted water (IPW), cultivation of polluted soil (CPS), and application of cyanobacterial manure (ACM), respectively. ^c^ The same letter in each biomass index of one vegetable indicates no significant difference (*p* > 0.05).

**Table 4:** Estimated daily intake (*EDI*) of MC-LR and risk quotient (*RQ*) via consuming the vegetables.

Vegetable	Treatment	*EDI* (**μg/kg/d**)	*RQ*	Risk Level ^a^
*Ipomoea batatas* L.	IPW	0.013 ± 0.003 a ^b^	0.32 ± 0.08 a	Medium risk
	CPS	0.019 ± 0.002 a	0.47 ± 0.04 a	Medium risk
	ACM	0.022 ± 0.008 a	0.55 ± 0.20 a	Medium risk
*Brassica juncea* L.	IPW	0.002 ± 0.000 a	0.06 ± 0.008 a	Low risk
	CPS	0.002 ± 0.001 a	0.05 ± 0.016 a	Low risk
	ACM	0.003 ± 0.001 a	0.09 ± 0.022 a	Low risk
*Brassica alboglabra* L.	IPW	0.004 ± 0.003 ab	0.10 ± 0.01 ab	Low risk
	CPS	0.001 ± 0.000 a	0.02 ± 0.01 a	Low risk
	ACM	0.004 ± 0.001 b	0.11 ± 0.01 b	Medium risk

^a^ Risk level was evaluated based on *RQ* value, i.e., high health risk (*RQ* > 1), moderate health risk (0.1 ≤ *RQ* ≤ 1), low health risk (*RQ* < 0.1). ^b^ The same letter in each biomass index of one vegetable indicates no significant difference (*p* > 0.05).

## Supplementary Materials

The following are available online at https://www.mdpi.com/2072-6651/12/8/523/s1, Table S1: Pearson’s correlation coefficients among MC-LR concentration, MDA contents and biomass of *Ipomoea batatas* L. (*n* = 12), Table S2: Pearson’s correlation coefficients among MC-LR concentration, MDA contents and biomass of *Brassica juncea* L. (*n* = 12), Table S3: Pearson’s correlation coefficients among MC-LR concentration, MDA contents and biomass of *Brassica alboglabra* L. (*n* = 12), Table S4: Water contents (%) of the tested vegetables, Table S5: Spiked recoveries (%, *n* = 4) of MC-LR at 5 ng/g in the tested vegetables and corresponding soil.
