# Supplementary material for: Bioaccumulation and Phytotoxicity and Human Health Risk from Microcystin-LR under Various Treatments: A Pot Study
Source: Toxins (Basel). 2020 Aug 14;12(8):523. doi: 10.3390/toxins12080523 (PMC7472386; doi:10.3390/toxins12080523)
Supplement: Supplementary file 1 [file toxins-12-00523-s001.pdf]

Supplementary Materials: Bioaccumulation and Phytotoxicity and Human Health Risk from Microcystin-LR under Various Treatments: A Pot Study

Lei Xiang, Yan-Wen Li, Zhen-Ru Wang, Bai-Lin Liu, Hai-Ming Zhao, Hui Li, Quan-Ying Cai, Ce-Hui Mo and Qing X. Li

Table S1. Pearson's correlation coefficients among MC-LR concentration, MDA contents and biomass of *Ipomoea batatas* L. (n = 12).

	RC [a]	SC	LC	RM	SM	LM	MR	PH	TW	AW
RC	-	0.548 *,[b]	0.161	0.118	0.476	0.700 *	−0.566 *	−0.803 **	−0.147	−0.098
SC	-	-	0.367	0.104	0.858 **	0.833 **	−0.889 **	−0.672 *	−0.538 *	−0.501
LC	-	-	-	0.421	0.457	0.617 *	−0.305	0.058	0.231	0.175
RM	-	-	-	-	0.478	0.276	−0.106	−0.125	0.441	0.401
SM	-	-	-	-	-	0.787 **	−0888 **	−0.563	−0.306	−0.343
LM	-	-	-	-	-	-	−0.767 **	−0.584 *	−0.265	−0.266

[a] RC, SC, LC indicate MC-LR concentration in root, stem, and leaf, respectively; RM, SM, and LM indicate MDA contents in root, and stem, and leaf, respectively; MR, PH, TW, and AW indicate main root length, plant height, total weight, and aerial part weight, respectively. [b] "*" and "**" indicate $p < 0.05$ and $p < 0.01$, respectively.

Table S2. Pearson's correlation coefficients among MC-LR concentration, MDA contents and biomass of *Brassica juncea* L. (n = 12).

	RC [a]	SC	LC	RM	SM	LM	MR	PH	TW	AW
RC	-	0.829**,[b]	0.558 *	−0.661 *	0.054	0.519 *	−0.520	−0.645 **	−0.663 *	−0.768 **
SC	-	-	0.284	−0.310	0.078	0.396 **	−0.342	−0.381	−0.316	−0.648 *
LC	-	-	-	−0.311	0.134	0.413	−0.297	0.517	−0.672 *	0.539 *
RM	-	-	-	-	0.199	−0.175	−0.048	0.270	0.476	0.463
SM	-	-	-	-	-	−0.559	0.032	0.014	0.120	−0.389
LM	-	-	-	-	-	-	−0.519	−0.687 *	−0.653 *	−0.343

[a] RC, SC, LC indicate MC-LR concentration in root, stem, and leaf, respectively; RM, SM, and LM indicate MDA contents in root, and stem, and leaf, respectively; MR, PH, TW, and AW indicate main root length, plant height, total weight, and aerial part weight, respectively. [b] "*" and "**" indicate $p < 0.05$ and $p < 0.01$, respectively.

Table S3. Pearson's correlation coefficients among MC-LR concentration, MDA contents and biomass of *Brassica alboglabra* L. (*n* = 12).

	RC [a]	SC	LC	RM	SM	LM	MR	PH	TW	AW
RC	-	0.960 **,[b]	0.435	0.608 *	0.685 *	0.847 **	−0.961 **	−0.347	−0.764 **	−0.674 *
SC	-	-	0.426	0.583 *	0.559 *	0.746 *	−0.917 **	−0.401	−0.692*	−0.600 *
LC	-	-	-	0.007	0.437	0.574 *	−0.240	−0.392	0.187	0.285
RM	-	-	-	-	−0.029	0.554 *	−0.594 *	−0.269	−0.571 *	−0.578 *
SM	-	-	-	-	-	0.658 *	−0.703 *	−0.058	−0.550	−0.412
LM	-	-	-	-	-	-	−0.790 **	−0.303	−0.555 *	−0.440

[a] RC, SC, LC indicate MC-LR concentration in root, stem, and leaf, respectively; RM, SM, and LM indicate MDA contents in root, and stem, and leaf, respectively; MR, PH, TW, and AW indicate main root length, plant height, total weight, and aerial part weight, respectively. [b] "*" and "**" indicate $p < 0.05$ and $p < 0.01$, respectively.

Table S4. Water contents (%) of the tested vegetables.

Vegetable	Root	Stem	Leaf
Ipomoea batatas L.	89.5 ± 4.7	88.7 ± 2.3	88.3 ± 0.9
Brassica juncea L.	86.5 ± 2.1	95.0 ± 2.1	93.6 ± 1.1
Brassica alboglabra L.	70.9 ± 25.2	92.2 ± 2.5	89.5 ± 1.2

Table S5. Spiked recoveries (%, *n* = 4) of MC-LR at 5 ng/g in the tested vegetables and corresponding soil.

Vegetable	Root		Stem		Leaf		Soil	
	Re [a]	RSD	Re	RSD	Re	RSD	Re	RSD
Ipomoea batatas L.	107.2	3.4	87.3	7.8	61.3	5.5	72.6	8.9
Brassica juncea L.	92.2	6.1	80.5	4.7	65.6	3.8	80.5	2.5
Brassica alboglabra L.	88.5	8.8	79.5	6.5	75.5	7.8	97.4	9.5

[a] "RE" and "RSD" indicate recovery and relative standard deviation, respectively.
